# Editorial: Studying novel immune signatures, targets, and drugs in hepatobiliary tumors: based on advanced technologies

**DOI:** 10.3389/fimmu.2023.1322220

**Published:** 2023-11-13

**Authors:** Zixue Xuan, Jian Song, Quanjun Yang, Ping Huang

**Affiliations:** ^1^ Center for Clinical Pharmacy, Cancer Center, Department of Pharmacy, Zhejiang Provincial People’s Hospital (Affiliated People’s Hospital), Hangzhou Medical College, Hangzhou, Zhejiang, China; ^2^ Key Laboratory of Endocrine Gland Diseases of Zhejiang Province, Zhejiang Provincial People’s Hospital (Affiliated People’s Hospital), Hangzhou Medical College, Hangzhou, Zhejiang, China; ^3^ Institute of Physiological Chemistry and Pathobiochemistry, University of Muenster, Muenster, Germany; ^4^ Department of Pharmacy, Shanghai Sixth People’s Hospital Affiliated Shanghai Jiao Tong University School of Medicine, Shanghai, China

**Keywords:** hepatocellular carcinoma, immune microenvironment, N6-methyladenosine (m 6 A), sphingolipids, metabolic reprogramming

Hepatobiliary tumors are among the most common malignancies occurring worldwide. Early diagnosis is difficult, and the clinical curative rate is low (1). Although the treatment of patients with hepatocellular carcinoma (HCC) has improved, their overall survival duration remains short (2). The diverse manifestations and the heterogeneity of HCC are important reasons for the difficulty in early detection, resulting in late treatment (3). Therefore, it is important to explore the intrinsic characteristics of HCC and identify new predictive biomarkers to improve the clinical efficacy and prognosis of patients with HCC.

With the development of precision medicine, especially the introduction of advanced technology, the molecular mechanisms underlying the occurrence and development of hepatobiliary tumors have gradually been unveiled. For example, in diagnostics, the Food and Drug Administration (FDA) has approved Foundation One CDx (324 genes) (4), based on second-generation sequencing, for companion diagnostics across multiple cancer types, multiple sites, and multiple drugs, and MSK-IMPACT (468 genes) (5), for complementary diagnosis of tumors. A review by Jiang et al. summarized the unique immune microenvironment of HCC and discussed the role of immunocompetent cells such as Kupffer cells (KCs), tumor-infiltrating lymphocytes, and cytotoxic T lymphocytes, along with the role of the major cytokines in HCC development.

Exosomes, an important type of extracellular vesicle (EV), play a role in intercellular communication through the proteins, nucleic acids, lipids, and metabolites they carry and are involved in several physiological and pathological processes, such as immune responses and cancer progression (6). Owing to their potential as drug delivery vehicles, exosomes have received considerable attention from researchers and pharmaceutical giants (6, 7). Li et al. highlighted the role of exosomes in the innate and inhibitory adaptive immune microenvironments and summarized that engineered exosomes may have good therapeutic effects that are targeted, along with low immunogenicity. Despite the promising potential of exosomes as therapeutic agents for HCC and the fact that many compounds target exosomal biogenesis, thus demonstrating their therapeutic potential in preclinical studies, clinical trials are lacking. Further studies are required to evaluate the clinical value and the adverse effects of these drugs. Given the role of exosomes in the tumor microenvironment, the combination of exosome-targeting compounds with other antitumor agents such as anti-PD-1/PD-L1 or chemotherapeutic agents may be a potential cancer treatment strategy.

In recent years, the pathogenesis of HCC has been gradually revealed, including N6-methyladenosine (m6A) (8), sphingolipids (Zhang et al.), and amino acid metabolic reprogramming (9), which play important roles. Based on these mechanisms, it is important to develop effective prognostic prediction models for patients with HCC. Therefore, this study advances the development and validation of prognostic models.

m6A is the most common modification in higher biological mRNA and lncRNA. Ma et al. established a prognostic model based on m6A-related genes and found that this model could predict the overall survival of patients and their immune microenvironment characteristics. Multiple immunofluorescence assays were used to confirm that patients in the high-risk group of the m6A-related gene prognostic model had obvious immunosuppressive characteristics, with massive infiltration of M2-polarized macrophage and T-regulatory cells. Previous studies have confirmed that sphingolipids play a crucial role in regulating HCC and that targeting and modulating sphingolipid synthesis may promote or inhibit HCC. A recent study by Zhang et al. showed that a six-gene signature can help select the appropriate treatment strategy and evaluate the characteristics of the immune microenvironment. In recent years, several studies have highlighted the involvement of metabolic reprogramming in the development of HCC. The study of metabolic reprogramming in HCC is of great importance to elucidate the metabolic regulation mechanisms of HCC and to explore corresponding target therapies. Recently, it has been reported that, during HCC progression, the pituitary tumor transforming gene 1 (PTTG1) can upregulate asparagine metabolism mediated by asparagine synthase (ASNS) and that the mTOR pathway is activated to promote the proliferation and progression of HCC (10). Amino acid metabolic reprogramming occurs when cells alter the metabolic pattern of one or more amino acids to meet the nutritional requirements for rapid proliferation that can manifest in the deficiency or accumulation of certain amino acids in tumor cells and the tumor microenvironment. The identification of amino acid metabolism-related genes and the establishment of a signature to effectively predict prognosis bear research significance. Li et al. found two molecular subtypes, of HCC, that were significantly enriched in amino acid metabolism-related signaling pathways. They constructed a prediction model and found that different subgroups have different probabilities of gene mutations. Patients with high-risk scores show a poor prognosis and are less sensitive to chemotherapy and targeted drugs. Since the maintenance of stemness features in HCC requires metabolic reprogramming, combining metabolic alterations with stemness features may predict the prognosis and immunotherapy response of patients with HCC. Wang et al. constructed a new stemness-metabolism-related model comprising sex, age, TNM staging, and gene signatures (PFKP, PDE2A, and UGT1A5) and found its high predictive value.

This study summarizes the heterogeneity of the immune microenvironment in HCC and establishes several prognostic models with good clinical application potential. Important aspects of this Research Topic have not been covered and merit further study. For example, all predictive models need to be validated in large clinical trials. Additionally, identifying potential immune-promoting or immunosuppressive subgroups require further research. The immune microenvironment may affect the therapeutic effect of immune checkpoint inhibitors (ICBs). Dissection of the tumor ecosystem of liver cancer, especially the tumor microenvironment and immunophenotype of patients with recurrent and drug-resistant disease, can deepen the understanding of the immune escape mechanism. Using the single-cell atlas of relapsed or drug-resistant liver cancer to study cell components and their interactions in the tumor microenvironment at the single-cell resolution level and understanding the mechanisms associated with tumor development may help to discover more effective immunotherapy strategies of HCC.

## Author contributions

ZX: Writing – original draft. JS: Writing – review & editing. QY: Supervision, Writing – review & editing. PH: Writing – original draft.
